# Ectopic expression of a cytochrome P450 monooxygenase gene *PtCYP714A3* from *Populus trichocarpa* reduces shoot growth and improves tolerance to salt stress in transgenic rice

**DOI:** 10.1111/pbi.12544

**Published:** 2016-03-11

**Authors:** Cuiting Wang, Yang Yang, Haihai Wang, Xiaojuan Ran, Bei Li, Jiantao Zhang, Hongxia Zhang

**Affiliations:** ^1^ National Key Laboratory of Plant Molecular Genetics Shanghai Institute of Plant Physiology and Ecology Chinese Academy of Sciences Shanghai China

**Keywords:** cytochrome P450 monooxygenase, development, PtCYP714A3, transgenic plant, salinity

## Abstract

In *Arabidopsis thaliana* and *Oryza sativa*, the cytochrome P450 (CYP) 714 protein family represents a unique group of CYP monooxygenase, which functions as a shoot‐specific regulator in plant development through gibberellin deactivation. Here, we report the functional characterizations of *PtCYP714A3*, an *OsCYP714D1/Eui* homologue from *Populus trichocarpa*. *PtCYP714A3* was ubiquitously expressed with the highest transcript level in cambium–phloem tissues, and was greatly induced by salt and osmotic stress in poplar. Subcellular localization analyses indicated that PtCYP714A3‐YFP fusion protein was targeted to endoplasmic reticulum (ER). Expression of *PtCYP714A3* in the rice *eui* mutant could rescue its excessive‐shoot‐growth phenotype. Ectopic expression of *PtCYP714A3* in rice led to semi‐dwarfed phenotype with promoted tillering and reduced seed size. Transgenic lines which showed significant expression of *PtCYP714A3* also accumulated lower GA level than did the wild‐type (WT) plants. The expression of some GA biosynthesis genes was significantly suppressed in these transgenic plants. Furthermore, transgenic rice plants exhibited enhanced tolerance to salt and maintained more Na^+^ in both shoot and root tissues under salinity stress. All these results not only suggest a crucial role of *PtCYP714A3* in shoot responses to salt toxicity in rice, but also provide a molecular basis for genetic engineering of salt‐tolerant crops.

## Introduction

As a class of important plant hormones, gibberellins (GAs) play crucial roles in promoting seed germination, stem elongation, leaf expansion and flower development (Eriksson *et al*., 2006; King *et al*., 2001; Ogawa *et al*., 2003; Schwechheimer, 2008). In higher plants, bioactive GA levels can be regulated via controlling the biosynthesis, deactivation and signal transduction of GAs. The biosynthesis of bioactive GAs such as GA_1_ and GA_4_ was initiated from geranylgeranyl diphosphate (GGDP), and catalysed by three types of enzymes including plastid‐localized terpene cyclases, membrane‐bound cytochrome P450 monooxygenases (P450s) and soluble 2‐oxoglutarate‐dependent dioxygenases (2ODDs) (Yamaguchi, 2008). The deactivation of GAs also involves several different mechanisms. The major enzymes responsible for the deactivation of bioactive GAs (GA_1_ and GA_4_) and their immediate precursors (GA_20_ and GA_9_) are GA2‐oxidases (GA2ox) that add a hydroxyl group to the C‐2 position of the substrates (Olszewski *et al*., 2002; Rieu *et al*., 2008; Thomas *et al*., 1999).

In *Arabidopsis*, AtGA2ox7 and AtGA2ox8 can inactivate the earlier GA biosynthetic intermediates, but cannot deactivate the bioactive GAs lacking C‐20 via 2‐hydroxylation (Lee and Zeevaart, 2005; Schomburg *et al*., 2003). GA methyltransferase (GAMT1 and GAMT2)‐mediated methylation, which is regulated by developmental stimuli, is another route to inactivate bioactive GAs (Varbanova *et al*., 2007). In rice, a cytochrome P450 monooxygenase CYP714D1 encoded by *Eui* gene that was cloned from the recessive tall rice mutant *elongated uppermost internode* (*eui*) can deactivate non‐13‐hydroxylated GAs (GA_4_, GA_9_ and GA_12_) by the 16α, 17‐epoxidation (Luo *et al*., 2006; Zhu *et al*., 2006). Although the 16α, 17‐[OH]_2_‐GAs were found in many other plant species, such as *Cibotium glaucum* (Yamane *et al*., 1988), *Lupinus albus* (Gaskin *et al*., 1992), *Malus domestica* (Hedden *et al*., 1993), *Pisum sativum* (Santes *et al*., 1995), *Prunus avium* (Blake *et al*., 2000) and *Populus trichocarpa* (Pearce *et al*., 2002),no any gene as yet was reported having similar functions to *Eui* gene in these species. Recently, two other *CYP714* gene family members, *CYP714B1* and *CYP714B2*, were found to encode GA13‐oxidases that negatively regulate shoot growth and participate in GA homoeostasis in rice (Magome *et al*., 2013). In *Arabidopsis*, two *Eui*‐like genes (*ELA1/CYP714A1* and *ELA2/CYP714A2*) encode Eui homologues that subtly regulate plant growth most likely through catalysing the deactivation of bioactive GAs similar to rice Eui (Zhang *et al*., 2011). Using a yeast expression system, CYP714A1 was revealed to be a GA‐deactivating enzyme that catalyses the conversion of GA_12_ to 16‐carboxylated GA_12_ (16‐carboxy‐16β, 17‐dihydro GA_12_), while CYP714A2 acts as a 13‐oxidase or 12α‐oxidase of GAs or GA precursors depending on the substrate (Nomura *et al*., 2013). All these reports suggest that *CYP714* gene family members from different plant species might have different functions in GA metabolic pathway.

The endogenous GA levels are also affected by environmental stimuli (Huang *et al*., 2009a; Nelson *et al*., 2009). Under salt stress condition, growth inhibition is the primary response of plants and may be the result of positive adaptation mechanisms (Munns, 2002). In *Arabidopsis,* DELLA proteins were found to integrate responses to independent hormonal and environmental signals of adverse conditions, and confer growth restraint upon salinity treatment (Achard *et al*., 2006). Recently, DWARF AND DELAYED FLOWERING 1 (DDF1), a salinity‐responsive AP2 transcription factor belonging to the DREB1/CBF subfamily, was reported to bind DRE‐like motifs in *GA2ox7* promoter and activate the expression of *GA2ox7*, leading to growth repression for stress adaptation (Magome *et al*., 2004, 2008). *DDF1*‐overexpressing *Arabidopsis* showed increased tolerance to cold, drought and heat stresses (Kang *et al*., 2011). In addition, gibberellins were suggested to be involved in *Arabidopsis* mitochondrial phosphate transporter (AtMPT)‐mediated early response to salt stress (Zhu *et al*., 2012). However, the potential application of GA‐related genes to improve salt tolerance in other plants including important crops and trees is barely reported (Shan *et al*., 2014).

Although the functions of some *CYP714* gene family members in *Arabidopsis* and rice have been studied, very limited information is known regarding these enzymes from other plant species. With the finish of genome sequencing and availability of developing genomic tools, *Populus* has been taken as an ideal model plant for trees (Jansson and Douglas, 2007; Tuskan *et al*., 2006). Previously, we investigated the function of rice *CYP714D1* gene in *Populus* (Wang *et al*., 2013). In this work, the role of *PtCYP714A3* gene from *Populus* in plant development and salt stress adaptation was investigated. We found that *PtCYP714A3* was a functional homologue of *OsCYP714D1*/*Eui* by complementation tests in the rice *eui* mutant and showed similar but not exactly the same functions in shoot development as did *OsCYP714D1*/*Eui*. Further study revealed that *PtCYP714A3* regulated Na^+^/K^+^ homoeostasis in transgenic rice plants to support their survival under salinity stress. Our results suggest that *PtCYP714A3* exerts distinct functions from *OsCYP714D1*/*Eui* in plant growth, and plays important roles in plant salt resistance.

## Results

### PtCYP714A3 encodes a putative cytochrome P450 monooxygenase in *Populus*

**Figure 1:**
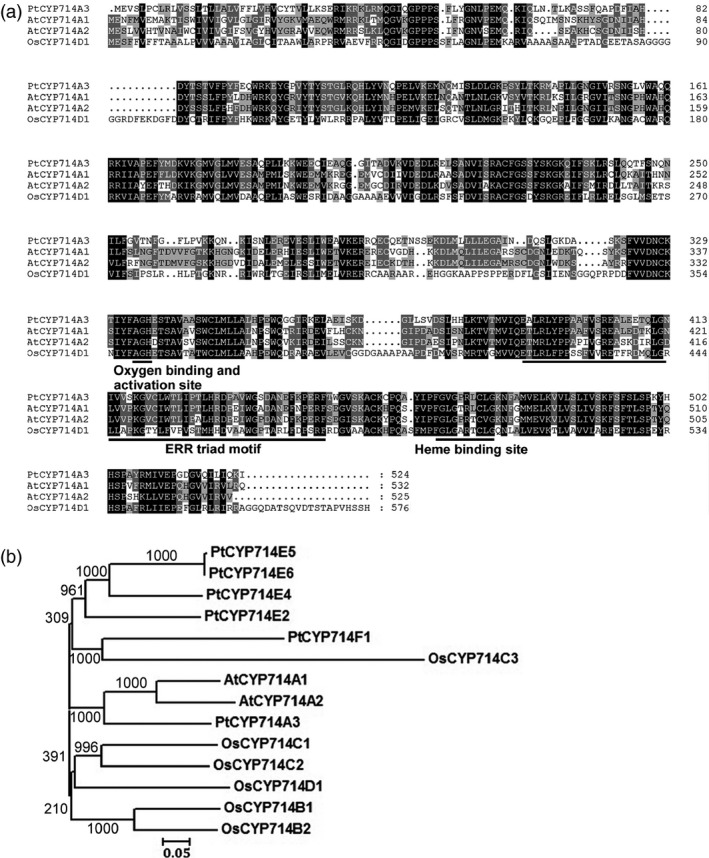
Amino acid sequence alignment and phylogenetic tree of different CYP714 protein family members. (a) Multiple alignment of the deduced amino acid sequences of CYP714 proteins from *Arabidopsis* (AtCYP714A1/A2) and rice (OsCYP714D1). (b) Phylogenetic tree of typical CYP714 proteins in *Populus*, *Arabidopsis* and *Oryza sativa* with bootstrap values conducted with Clustal X and Mega 3 program. The GenBank accession numbers for *Populus* CYP714 genes are as follows: *PtCYP714A3* (XM_00631280.1), *PtCYP714E2* (XM_006375124.1), *PtCYP714E4* (XM_002319405.2), *PtCYP714E5* (XM_002311935.2) and *PtCYP714F1* (XM_002314788.1).

A BLAST search of the cytochrome P450 homepage (http://drnelson.uthsc.edu/ CytochromeP450.html) resulted in the identification of six homologues in the *Populus* genome, designated as PtCYP714A3 (Potri.019G064600.1), PtCYP714E2 (Potri014G052000.1), PtCYP714E4 (Potri.013G160800.1), PtCYP714E5 (Potri.008G026300.1), PtCYP714E6 (Potri.008G026200.1) and PtCYP714F1 (Potri.010G116300.1). These proteins share 63.33% identity and 69.99% similarity, with PtCYP714E6 as a redundant duplicate of PtCYP714E5 (99% identity). Among these proteins, PtCYP714A3 shares the highest sequence identity with AtCYP714A1 (58.99%), AtCYP714A2 (53.20%) from *Arabidopsis* and OsCYP714D1 (41.35%) from rice, respectively (Figure 1a,b). Similar to AtCYP714A1, AtCYP714A2 and OsCYP714D1, PtCYP714A3 is characterized by an oxygen binding and activation site, a ERR triad motif and a Haeme binding site, as a common feature of CYP714 subfamily. The fact that *Arabidopsis* genome encodes only two CYP714 members suggests that *Populus* could have duplicated this class of gene during its long evolution for better adaptation to the unstable environments over a long lifespan.

### Expression pattern of *PtCYP714A3* in *Populus*

**Figure 2:**
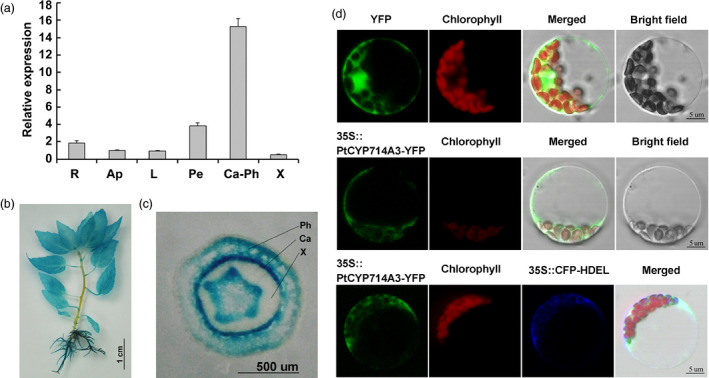
Expression pattern and subcellular localization of PtCYP714A3 in *populus*. (a) Relative expressional levels of *PtCYP714A3* gene in different tissues of *Populus trichocarpa* (Torr. & Gray) genotype Nisqually‐1. R, Root; Ap, apical bud; L, leaf; Pe, petiole; Ca‐Ph, cambium–phloem zone; X, xylem. the expression value of *PtCYP714A3* in root was set to 1. Error bars are means ± SD of three biological replicates. The experiment was repeated two times independently. (b, c) Histochemical GUS analysis of a *PtCYP714A3* promoter–GUS transgenic plant and a transverse section of the stem. (d) Subcellular localization of PtCYP714A3. Confocal laser scanning microscopy images of poplar mesophyll protoplasts transiently expressing yellow fluorescent protein (YFP), or PtCYP714A3‐YFP alone and cyan fluorescent protein (CFP)‐HDEL together under the control of the 35S promoter were shown.

To elucidate the possible roles of *PtCYP714A3*, we first performed quantitative real‐time PCR and investigated its expression pattern in *Populus trichocarpa* (Torr. & Gray) genotype Nisqually‐1. We observed that *PtCYP714A3* was predominantly expressed in the cambium–phloem tissues than in the other tissues such as roots, shoot apexes, leaves, petioles and xylem tissues (Figure 2a). We also generated *PtCYP714A3* promoter–GUS reporter vector and introduced it into Shanxin yang. Consistent with the qRT‐PCR results, GUS was ubiquitously expressed in various tissues (Figure 2b), with a strong GUS expression in the cambium zone of stem (Figure 2c).

### PtCYP714A3 is targeted to endoplasmic reticulum (ER)

Cellular and subcellular localization of a certain protein may indicate how and/or where it works. Previous studies have shown that OsCYP714D1/Eui, AtCYP714A1 and AtCYP714A2 are all targeted to ER (Zhang *et al*., 2011; Zhu *et al*., 2006). To determine the subcellular localization of PtCYP714A3, we transiently expressed PtCYP714A3‐YFP (yellow fluorescent protein) fusion protein in poplar leaf protoplasts. HDEL, an ER‐localized reporter (Dong *et al*., 2008), was also co‐transformed with PtCYP714A3‐YFP to verify the ER targeting of PtCYP714A3 protein. As we have expected, the fluorescence of PtCYP714A3‐YFP overlapped perfectly with that of CFP‐HDEL, revealing that PtCYP714A3‐YFP fusion protein was indeed targeted to the ER organella (Figure 2d).

### *PtCYP714A3* can functionally complement the rice *eui* mutant

**Figure 3:**
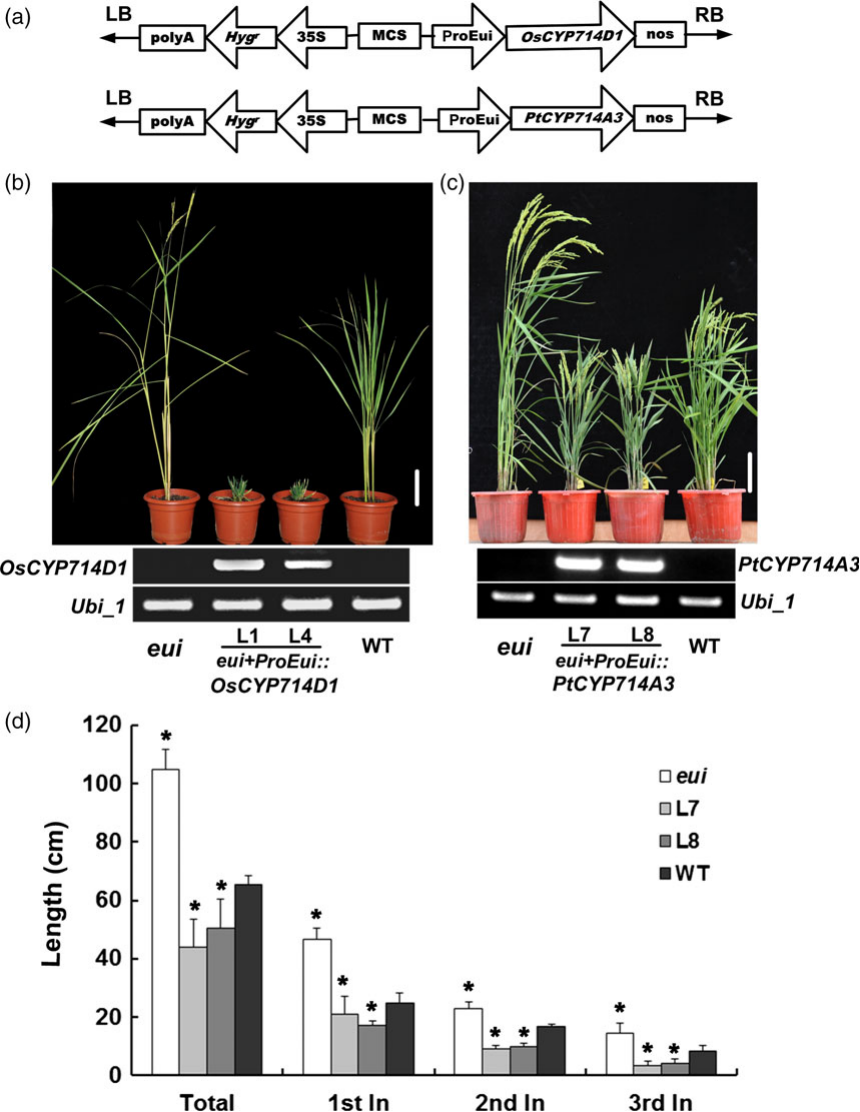
Genetic complementation of rice *eui* mutant by *PtCYP714A3*. (a) Schematic representation of T‐DNA region transformed into *eui* mutant. (b) Growth phenotypes of WT, *eui* and two *OsCYP714D1* complementary lines. *eui, elongated uppermost internode* rice mutant; L1 and L4, independent *eui* transgenic lines expressing *OsCYP714D1/Eui*cDNA under the control of *Eui* promoter; WT, wild type. Bar = 10 cm. (c) Growth phenotypes of WT, *eui* and two *PtCYP714A3* complementary lines. L7 and L8, independent *eui* transgenic lines expressing *PtCYP714A3* cDNA under the control of *Eui* promoter. Bar = 10 cm. (d) Plant heights of *eui* mutant, wild‐type and the *PtCYP714A3* transgenic plants. Values are means ± SD from 30 individual plants with three independent biological replicates. * indicates significant difference in comparison with the WT at *P* < 0.05 (Student's *t*‐test).

The rice *eui* mutant displays excessive‐shoot‐growth phenotype (Zhu *et al*., 2006). To understand whether *PtCYP714A3* might have similar functions as the rice ortholog, we heterologously expressed *PtCYP714A3* driven by the *Eui* promoter in the *eui* background (Figure 3a). In parallel, *OsCYP714D1/Eui* driven by the *Eui* promoter was separately introduced into *eui* mutant as well (Figure 3a). At least ten independent transgenic lines were obtained for each gene. RT‐PCR analyses confirmed the expression of *OsCYP714D1/Eui* or *PtCYP714A3* in the *eui* mutant, respectively (Figure 3b,c). Compared to the wild‐type and *eui* mutant plants, all transgenic lines (L1 and L4) expressing *OsCYP714D1/Eui* showed severely stunted growth, and failed to flower and produce seeds (Figure 3b), whereas those expressing *PtCYP714A3* only showed semi‐dwarfed growth and set seeds successfully (Figure 3c). Detailed studies with the T_4_ seeds of two *PtCYP714A3* transgenic lines (L7 and L8) showed that the semi‐dwarfed phenotype in *PtCYP714A3* transgenic plants was attributed to the shortened upper most (1st), the second and the third internodes (Figure 3d). These results suggest that *PtCYP714A3* biologically complemented the excessive‐shoot‐growth phenotype of *eui* mutant, but did not work exactly the same as did *OsCYP714D1*.

### Expression of *PtCYP714A3* inhibits shoot growth and promotes tillering in transgenic rice plants

**Figure 4:**
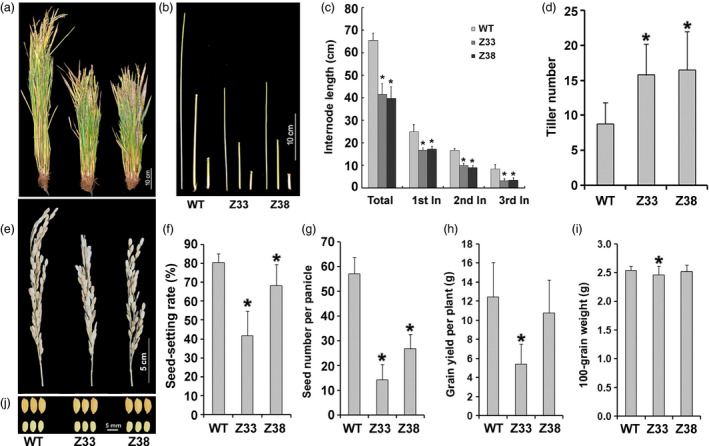
Expression of *PtCYP714A3* inhibits shoot growth and promotes tillering in transgenic rice plants. (a) Field‐grown plants. (b, c) Internode lengths. (d) Tiller numbers. (e) Mature panicles. (f) Seed‐setting rates. (g) Seed numbers per panicle. (h) Grain yields per plant. (I) One‐hundred‐grain weights. (j) Seeds with and without seed coat. WT, wild type; Z33 and Z38, independent transgenic lines. Values are means ± SD from 30 individual plants with three independent biological replicates. * indicates significant difference in comparison with the WT at *P* < 0.05 (Student's *t*‐test).

To investigate the exact function of PtCYP714A3 in plant, we introduced the construct containing the coding sequence of *OsCYP714D1/Eui* or *PtCYP714A3*, driven by the *Eui* promoter, into the genome of rice (ZH11) by *Agrobacterium*‐mediated transformation (Figure 3a). More than 10 independent transgenic lines were successfully obtained for each gene construct. The integration of *PtCYP714A3* into the rice genome was confirmed by PCR analyses (Figure S1a). Further analysis by RT‐PCR indicated the successful expression of *PtCYP714A3* in the selected transgenic rice plants (Figure S1b). Overexpression of *OsCYP714D1/Eui* led to severely dwarfed phenotype in all transgenic lines, just the same as its overexpression in the *eui* mutant (Figure S1c). However, all transgenic plants ectopically expressing *PtCYP714A3* showed consistent semi‐dwarfed phenotype regardless of the expression levels of transgene. Therefore, we selected two independent homozygous transgenic lines which showed high expression of *PtCYP714A3* (Z33, Z38) for subsequent phenotypic analyses (Figure S1c). Compared to WT plants, *PtCYP714A3* transgenic plants produced shorter shoots, including internodes and panicles (Figure 4a–c,e). In addition, increased tiller number of the transgenic lines was also a prominent difference from the WT (Figure 4d). To determine whether expression of *PtCYP714A3* would affect seed development in transgenic plants, wild‐type and homozygous T_4_ transgenic plants were chosen for field trial in 2013 (transgenic trial permit number: 2013‐T018). Compared to the wild‐type plants, both transgenic lines produced smaller panicles and seeds with decreased seed setting, leading to reduced grain yield per plant (Figure 4e–j; Table S2).

### Expression of *PtCYP714A3* reduces the contents of bioactive GAs in transgenic rice plants

**Figure 5:**
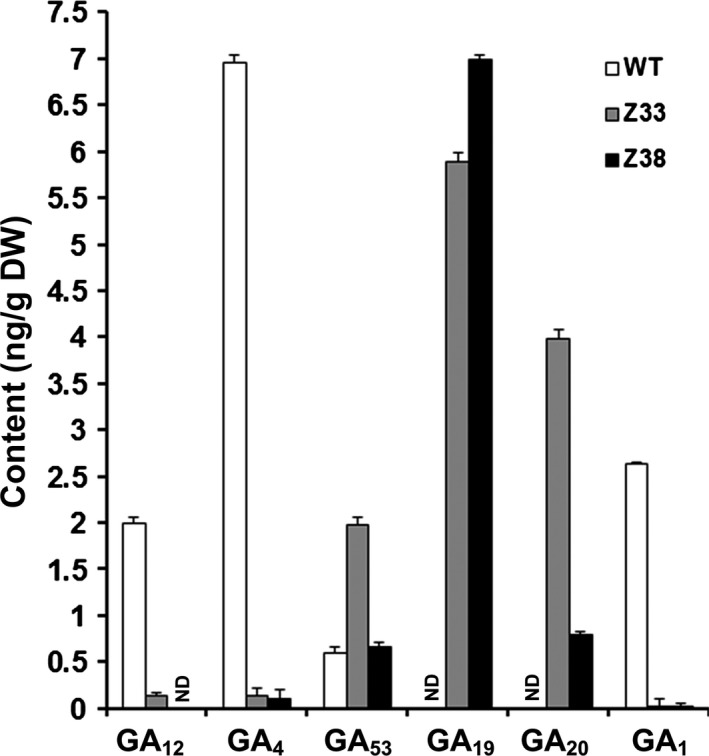
Endogenous GA levels in the stems of 2.5‐month‐old wild‐type and PtCYP714A3 transgenic plants. WT, wild type; Z33 and Z38, independent transgenic lines. Values are means ± SD of three biological replicates of ten individual plants from the WT or the transgenic lines. ND, not detected.

To understand whether *PtCYP714A3* indeed functions in GA deactivation, endogenous levels of GA precursors and bioactive GAs in the internodes of WT and *PtCYP714A3* transgenic plants were examined. Compared to the WT, the levels of both bioactive GAs (GA_1_ and GA_4_) and GA_12_, the common precursor in GAs biosynthesis pathway, were extremely lower or even undetectable in transgenic plants (Figure 5). On the contrary, the level of the other important precursor, such as GA_53_, was significantly higher in both transgenic lines, especially in line Z33 (Figure 5). We also determined several other immediate precursors of bioactive GAs. Both GA_19_ and GA_20_ were measurable in transgenic rice plants with the concentrations waving from 0.79 to 6.99 (ng/g), but they were undetectable in WT plants (Figure 5). GA_9_ was too low to be detected in both WT and transgenic plants (data not shown).

**Figure 6:**
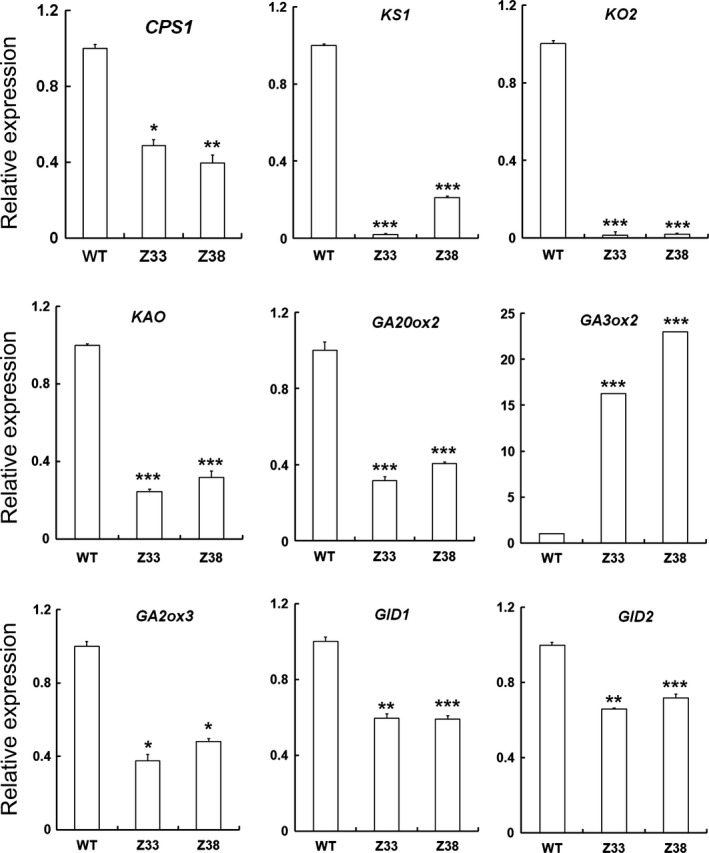
Relative expressional levels of GA‐related genes in wild‐type and PtCYP714A3 transgenic plants. WT, wild type; Z33 and Z38, independent transgenic lines. The name and the accession numbers of the genes are as the following: CPS1, *ent*‐copalyl diphosphate synthase (AP004572); KS1, *ent*‐kaurene synthase (OSJN00255); KO2, *eht*‐kaurene oxidase (AP005471); KAO, *ent*‐kaurenoic acid oxidase (AP000616); GA20ox2, GA 20‐oxidase (AB077025); GA3ox2, GA 3‐oxidase (AB056519); GA2ox3, GA 2‐oxidase (NM_001050827); GID1, soluble GA receptor (AB211399); GID2, F‐box protein (AB100246). Values are means ± SD of three biological replicates from the WT or the transgenic lines. Significant differences were analysed with Student's *t*‐test. *, *P* < 0.05; **, 0.05 < *P* < 0.01; ***, 0.01 < *P* < 0.001.

To explain why GA accumulation was affected in transgenic plants, we examined the expression levels of genes in GA biosynthesis, deactivation and signal pathways by quantitative real‐time PCR. Among these genes, the transcription levels of those involved in the synthesis of the common precursor GA_12_, such as *CPS*, *KS1*, *KO2* and *KAO*, all decreased significantly (Figure 6). GA20ox and GA3ox are the main enzymes that catalyse the syntheses of bioactive GAs (GA_1_ and GA_4_) (Itoh *et al*., 2001; Sasaki *et al*., 2002). We found that the expression level of *GA20ox2* decreased whereas that of *GA3ox2* increased, implying that they showed opposite variation in these transgenic plants. In addition, the transcription levels of the bioactive GA deactivation gene *GA2ox3* (Sakai *et al*., 2003), the GA receptor gene *GID1* (Ueguchi‐Tanaka *et al*., 2005) and the F‐box protein gene *GID2* (Sasaki *et al*., 2003) also decreased in transgenic rice compared with that in the WT plants (Figure 6). These results indicate that expression of *PtCYP714A3* influences GA accumulation and GA metabolic gene expression.

### Expression of *PtCYP714A3* confers salt tolerance on transgenic rice plants

**Figure 7:**
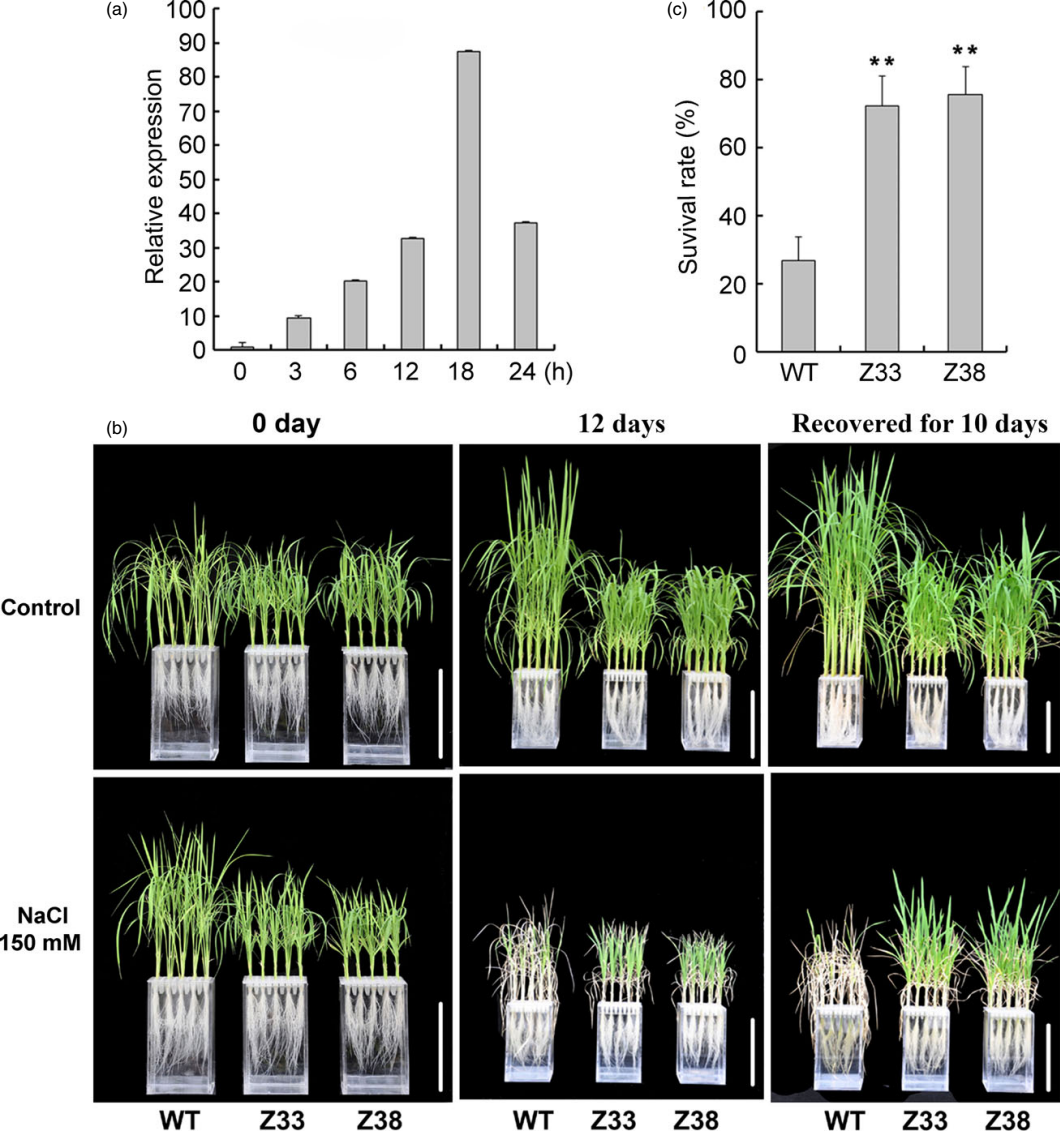
Expression *PtCYP714A3* in response to high‐salinity and salt stress tolerance analyses of transgenic rice plants. (a) Expression of *PtCYP714A3* is responsive to NaCl stress. Poplar leaves were cut into sections and divided into several groups that were treated separately with 200 mm NaCl for different time points. Samples were collected at 0, 3, 6, 12, 18 and 24 h after the initiation of treatment. Data represent the average of three independent experiments. (b, c) Phenotypes (b) and survival rates (c) of three‐week‐old seedlings of wild‐type and *PtCYP714A3* transgenic plants treated with 150 mm NaCl for 12 d followed by 10 days of recovery without NaCl. WT, wild type; Z33 and Z38, independent transgenic lines. Values are means ± SD (*n* = 4) from three independent experiments. Asterisks indicate statistically significant difference in comparison with the WT (Student's *t*‐test, **, *P* < 0.01).

Previous studies have shown that GAs also function in plant response to abiotic stress (Magome *et al*., 2004, 2008; Shan *et al*., 2014). To investigate the functions of *PtCYP714A3* and its potential value in improving salt tolerance in plants, we first extracted total RNA from the leaves of Shanxin yang treated with 150 mm NaCl for increasing periods of time and performed qRT‐PCR. We found that expression of *PtCYP714A3* was steadily induced by salt from 0 to 24 h, and reached a maximal 87‐fold induction at 18 h (Figure 7a). We then examined the effects of salt on the growth of transgenic plants at whole‐plant scale. Under normal growth condition, both WT and transgenic lines all grew well (Figure 7b). However, after the plants were treated with 150 mm NaCl for 12 days, obvious differences were observed between WT and transgenic plants. While WT plants became wilted, transgenic lines appeared to be less impaired by salt stress (Figure 7b). Although salt stress considerably affected the growth of all the plants in general, transgenic plants were remarkably more vigorous. Compared to WT, most *PtCYP714A3‐*expressing plants exhibited near‐to‐normal leaf colour and showed a superior survival rate at the end of treatment: more than 70% of *PtCYP714A3* transgenic, whereas less than 30% of WT plants survived (Figure 7c). All these results indicate that expression of *PtCYP714A3* in rice enhanced salt tolerance in transgenic plants.

### *PtCYP714A3* expression regulates tissue Na^+^ and K^+^ distribution

**Figure 8:**
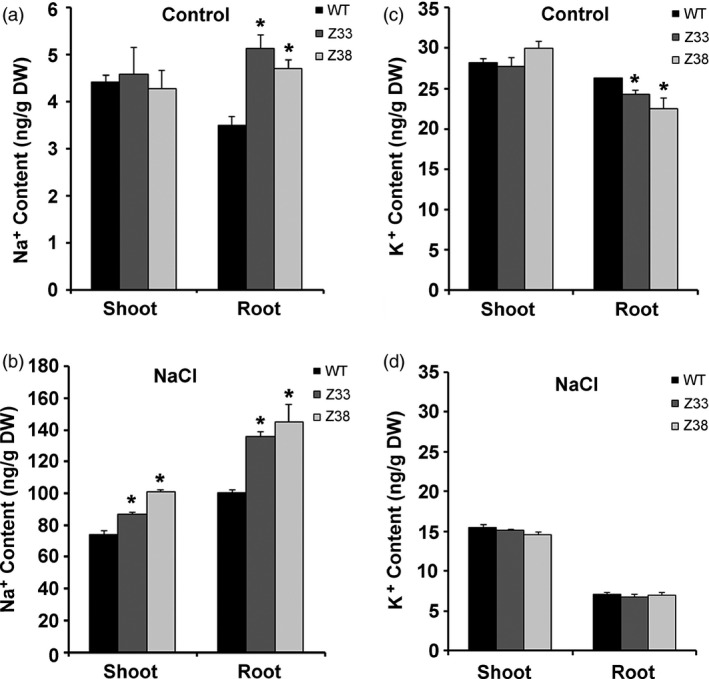
Na^+^ and K^+^ contents in roots and shoots of wild‐type (WT) and transgenic rice plants (Z33 and Z38). At the end of 0 or 150 mm NaCl treatment, plant materials were harvested and pooled into roots and shoots for the measurement of Na^+^ and K^+^ contents. (a) Na^+^ contents of different tissues at 0 mm NaCl. (b) Na^+^ contents of different tissues at 150 mm NaCl. (c) K^+^ contents of different tissues at 0 mm NaCl. (d) K^+^ contents of different tissues at 150 mm NaCl. Values are means ± SD (*n* = 4) from two independent experiments. Asterisks indicate statistically significant difference in comparison with the WT (Student's *t*‐test, *, *P* < 0.05).

To understand how the introduced *PtCYP714A3* gene improved salt‐tolerant capacity in transgenic plants, we examined the distribution of Na^+^ and K^+^ contents in different tissues of WT and transgenic rice plants after treated with or without 150 mm NaCl for 12 days. Under normal growth condition, WT showed less Na^+^ and more K^+^ content in the roots, although both WT and transgenic Z33, Z38 lines contained approximately equal Na^+^ and K^+^ content in the shoots (Figure 8a,c). Under salt stress condition, Na^+^ content increased in all plant tissues accompanied by a decrease in K^+^ content. However, Na^+^ content in Z33 and Z38 was significantly higher than that in WT in both shoots and roots (Figure 8b). Generally, WT and transgenic plants accumulated equivalent levels of K^+^ in both shoots and roots (Figure 8d). Taken together, our data suggest that *PtCYP714A3* regulates Na^+^ and K^+^ homoeostasis under salt stress condition in transgenic rice plants.

### Expression of salt stress‐related genes is affected in transgenic plants

**Figure 9:**
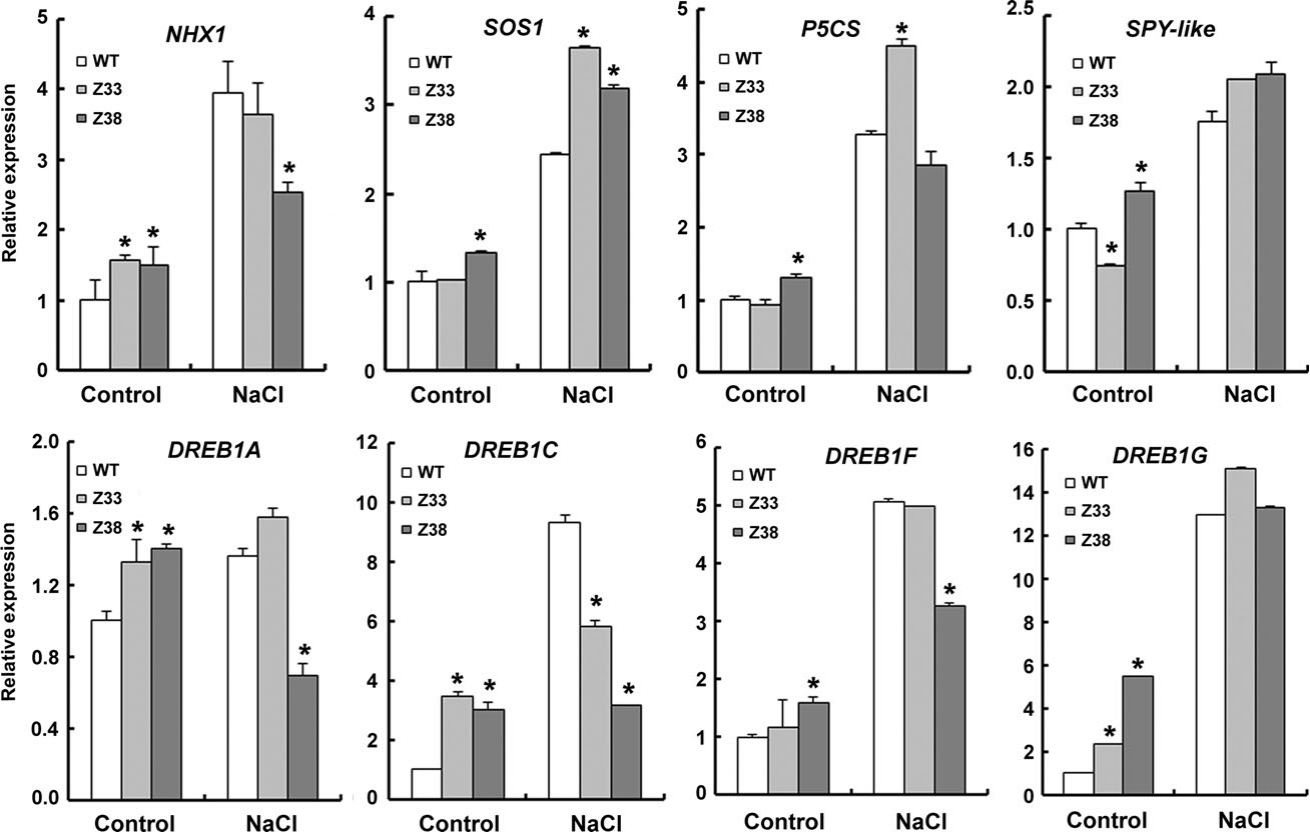
Quantitative real‐time PCR analyses of salt tolerance‐related marker genes. Three‐week‐old seedlings treated with 150 mm NaCl for 12 h or 0 h (Control) were harvested for total RNA extraction, transverse transcription and real‐time PCR analyses. WT, wild type; Z33 and Z38, independent transgenic lines. Values are means ± SD of three biological replicates from the WT or the transgenic lines. Asterisks indicate statistically significant difference in comparison with the WT (Student's *t*‐test, *, *P* < 0.05). The TIGR loci of these genes are as follows: *NHX1* (Os07g0600900); *SOS1* (Os12g0641100); *P5CS* (Os05g0455500); *SPY*‐*like* (Os08g0559300); *DREB1A* (Os09g35030); *DREB1F* (Os01g73770); *DREB2A* (Os01g07120).

To further investigate the role of PtCYP714A3 in salt stress adaptation of plants, the relative transcript levels of a series of salt stress‐related marker genes before or after NaCl stress treatment were analysed (Figure 9). *NHX1* and *SOS1* encode Na^+^/H^+^ the antiporters that compartment Na^+^ from cytoplasm into the vacuole (NHX1) or out of the plant cell (SOS1). The expression levels of *NHX1* and *SOS1* in transgenic rice (except *SOS1* in Z33) were slightly higher than in WT under nonstress condition, and increased significantly after salt stress in both WT and transgenic lines. Compared to *NHX1*, the expression increase of *SOS1* in transgenic plants was more significant than that in WT, indicating that in transgenic rice, more sodium ions were pumped out of the plant cells than were compartmented into the vacuole under salt stress. The enzyme of P5CS (Δ^1^‐pyrroline‐5‐carboxylate synthase) controls the rate‐limiting step of glutamate‐derived proline biosynthesis and is induced by high salt, dehydration, cold and ABA treatment (Igarashi *et al*., 1997). The transcription of *P5CS* was also induced by NaCl, and an almost fourfold increase was observed in Z33 upon NaCl treatment, much higher than that in WT (about 2 folds). The *SPY* gene encodes an *O*‐linked *N*‐acetylglucosamine transferase, a negative regulator of plant GA signalling, and is inducible by drought stress and slightly responsive to salt stress in *Arabidopsis* (Qin *et al*., 2011; Steiner *et al*., 2012). The expression of a homologous *SPY* in rice named *SPY‐like* was analysed in this study. The expression level of *SPY‐like* was slightly lower in Z33, but higher in Z38 than in WT before NaCl treatment, although no obvious difference was seen among these three lines after NaCl stress. DREBs (the dehydration‐responsive element‐binding proteins) are a kind of transcript factors in response to drought, high‐salt and cold stresses (Dubouzet *et al*., 2003; Wang *et al*., 2008; Mao and Chen 2012). So the expressions of *DREB1* genes in rice were analysed. All of these genes were induced to varying degrees by salt stress in the wild‐type rice. All these genes were expressed at higher levels in Z33 and Z38 than in WT under normal situations (except *DREB1F* in Z33). Under NaCl stress, the expression levels of *DREB1A*, *DREB1C* and *DREB1F* were decreased in Z38. All these results indicate that expression of *PtCYP714A3* altered the expression of salt stress‐related genes in transgenic plants.

## Discussion

Previous reports that CYP714D1/Eui functions as a GA 16α, 17‐epoxidase to inactivate GA_12_, GA_9_ and GA_4_ in rice suggest that 16α, 17‐epoxidation might be an important process of GA deactivation existed in a variety of plant species. Indeed, 16α, 17‐[OH]_2_‐GAs have been detected in many plant species including *Populus trichocarpa* (Blake *et al*., 2000; Gaskin *et al*., 1992; Hedden *et al*., 1993; Pearce *et al*., 2002; Santes *et al*., 1995; Yamane *et al*., 1988; Zhu *et al*., 2006). In our previous study, heterologous expression of *CYP714D1/Eui* gene led to improved growth rate and biomass of transgenic *Populus*, just opposite to the phenotypes of *CYP714D1*/*Eui*‐overexpressing rice (Wang *et al*., 2013). To understand these unexpected results, it is necessary to verify whether *Populus CYP714* gene family members have similar functions to *CYP714D1/Eui*. As *PtCYP714A3* has the maximum sequence similarity with *CYP714D1/Eui*, we cloned this gene and verified its possible biological function in transgenic rice plants. We show here that PtCYP714A3, a member of the P450 (CYP) 714 gene family, is involved in GA deactivation and salt resistance in rice.

PtCYP714A3 shares very high amino acid sequence identity with CYP714D1/Eui (OsCYP714D1), AtCYP714A1 and AtCYP714A2 that contain all the highly conserved domains (Figure 1a,b; Zhang *et al*., 2011; Zhu *et al*., 2006), indicating its possible role as a putative cytochrome P450 monooxygenase in *Populus*. As the genus *Populus* has become an ideal model plant for forest trees (Jansson and Douglas, 2007; Tuskan *et al*., 2006), a comparative study on the genetic functions of CYP714 gene family between rice and *Populus* could shoot inspective light into the difference in the mechanisms of plant growth and development under different environmental conditions between herbaceous and woody plants. *PtCYP714A3* was predominantly expressed in cambium–phloem cells, and its expression was strongly induced by salt stress in poplar (Figures 2a–c and 7a). Therefore, the high expression of PtCYP714A3 in cambium–phloem tissues of wild‐type poplar may imply a crucial role of PtCYP714A3 in GA metabolism and abiotic stress response in trees.

With a view to the high sequence homology of CYP714D1/Eui and PtCYP714A3, the biological role of PtCYP714A3 was investigated by *eui* mutant analyses (Figure 3a–d). The most dramatic phenotypic change in *eui* mutant was the elongated shoot growth, especially the uppermost internode. *PtCYP714A3* restored the growth phenotype of *eui* to the wild type, indicating that *PtCYP714A3* can be employed as a functional allele of *CYP714D1*/*Eui* in plants. However, a severely dwarfed phenotype was observed in the transgenic *eui* plants complemented with *CYP714D1/Eui* (Figure 3b–d). One explanation of this observation is that CYP714D1/Eui and PtCYP714A3 may have overlapped but not exactly the same function in rice and poplar. Another possibility is that CYP714D1/Eui is more powerful than PtCYP713A3 in controlling plant height. This postulation is also supported by the observation in transgenic poplar plants overexpressing PtCYP714A3, which showed no significant changes in the growth rate and biomass production (Figure S2a–e). More detailed molecular and biochemical studies will help to dissect the exact functions of both proteins in plants.

To clarify the exact biological functions of *PtCYP714A3*, we further ectopically expressed *PtCYP714A3* in ZH11 (Figures 4a–i and S1a–c), and examined the endogenous GA contents and the transcript levels of GA pathway‐related genes. We found that *PtCYP714A3* transgenic rice showed the most comparability with transgenic plants expressing *AtCYP714A2* (Nomura *et al*., 2013; Zhang *et al*., 2011). First, both *PtCYP714A3* and *AtCYP714A2* transgenic rice plants were semi‐dwarfed, with increased tillers (Figure 4a–d; Zhang *et al*., 2011). Second, the variation tendencies of most endogenous GA levels in these two kinds of transgenic plants were consistent. Based on the previous report, in *AtCYP714A2* transgenic plants, the levels of GAs in the non‐13‐hydroxylation pathway, including GA_12_, GA_15_, GA_24_ and GA_4_, all decreased, whereas those of 13‐hydroxy GAs, including GA_44_, GA_19_, GA_20_ and GA_1,_ all increased with the exception of GA_53_ which was unaffected (Nomura *et al*., 2013). In our study, most of detected GAs (GA_12_, GA_4_, GA_53_, GA_19_ and GA_20_) showed similar tendencies in *PtCYP714A3* transgenic rice, except for bioactive GA_1_, which decreased rather than increased (Figure 5). In addition, the expression level variations of GA pathway‐related genes in *PtCYP714A3*‐expressing plants sustained that PtCYP714A3 most likely possesses the function of AtCYP714A2. The expression of GA receptors (GID1), F‐box protein (GID2) as well as bioactive GA‐deactivating enzyme (GA2ox3) was all reduced along with decreased bioactive GAs (GA_1_ and GA_4_) (Figure 6). The transcript level of GA3ox which catalyses the conversion of GA_20_ and GA_9_ to bioactive GAs (GA_1_ and GA_4_) also increased. However, the transcript level of *GA20ox2* which encode the important GA‐oxidase involved in most steps of the bioactive GA biosynthesis pathway decreased, opposite to that of the *GA3ox2* gene (Figure 6). As the contents of GA_19_ and GA_20_, the intermediates produced in the 13‐hydroxylation (13‐H) pathway of GA biosynthesis, dramatically increased (Figure 5), we presumed that the inconformity between the expressions of *GA20ox2* and *GA3ox2* was attributed to the regulatory mechanism of intermediate substrates. Among the detected GA pathway genes, the most intriguing ones are those genes (*CPS*, *KS*, *KO* and *KAO*) participating in the biosynthesis of GA_12_, the common precursor of both 13‐H and non‐13‐H GA pathways. Expressions of all these genes reduced significantly, although the content of GA_12_ was extremely low or even undetectable in transgenic rice (Figures 5 and 6). In *Arabidopsis*, AtCYP714A2 was reported as a bifunctional enzyme that preferentially catalyses C‐12 hydroxylation of the *ent*‐gibberellane carbon skeleton, leading to the conversion of GA_12_ to 12α‐hydroxy GA_12_, and C‐13 hydroxylation of the *ent*‐kaurane carbon skeleton, by which steviol (*ent*‐13‐hydroxy asurenoic acid) was detected as the sole product when *ent*‐kaurenoic acid was added as a substrate (Nomura *et al*., 2013). As the content of GA_53_ was not affected along with the decrease of its immediate precursor GA_12_ in *PtCYP714A3* transgenic rice (Figure 5), and GA_53_ was also produced from steviol in *AtCYP714A2* transgenic plants (Nomura *et al*., 2013), we postulate that PtCYP714A3 might have similar function as AtCYP714A2 and could also catalyse the production of steviol‐like substance, and thus, the transcripts of *CPS*, *KS*, *KO* and *KAO* were inhibited by feedback regulation. Therefore, we deduced that, just similar to AtCYP714A2, PtCYP714A3 also functions directly in deactivating GA_12_ and converting *ent*‐kaurenoic acid into steviol, and thus plays an important role in regulating plant growth and development through fine‐tuning GA homoeostasis. However, due to the tremendous species differences between perennial woody *Populus* and annual herbaceous *Arabidopsis* or rice, delicate differences could exist among the CYP714 families members, including their expression patterns (Figure 2; Zhang *et al*., 2011), their functions on the metabolism of GA_1_ (Figure 5; Nomura *et al*., 2013; Zhang *et al*., 2011), and phenotypes in transgenic *Populus* and rice overexpressing *PtCYP714A3* (Figures 4, S1c and S2d).

Previous studies have suggested that GA2ox genes including *GA2ox7* were up‐regulated by high‐salinity stress in *Arabidopsis* (Magome *et al*., 2008). Overexpression of OsGA2ox5 or DDF1, an AP2 transcription factor of the DREB/CBF subfamily, activated the expression of *GA2ox7*, and enhanced the salt tolerance in transgenic plants (Magome *et al*., 2004, 2008; Shan *et al*., 2014). We found that expression of *PtCYP714A3* was also responsive to high‐salinity and osmotic stress (Figures 7a and S3a), and transgenic rice seedlings expressing *PtCYP714A3* showed improved tolerance to salt and osmotic stress, with higher survival rates and less growth inhibition, than did the wild‐type seedlings (Figures 7b,c and S3b,c).

Maintaining low levels of sodium ions in the cell cytosol is critical for plant growth and development. Salt‐tolerant plants usually had lower Na^+^ contents (Huang *et al*., 2009b; Tang *et al*., 2014). Under high‐salt stress condition, Na^+^ content in both shoots and roots of the *PtCYP714A3* transgenic rice was higher than that of in WT (Figure 8b). This could be due to the different functional mechanisms of *PtCYP714A3* and other salt‐resistant genes (Tang *et al*., 2014). Similar results were also observed in transgenic poplar plants overexpressing PtCLB10s which accumulated lower Na^+^ in leaves but higher Na^+^ in stems than did the WT plants (Tang *et al*., 2014). In addition, the semi‐dwarfed transgenic rice plants might have higher concentration ratio of sodium ion than the wild type.

To further understand the salt‐tolerant mechanism in *PtCYP714A3*‐overexpressing plants, we compared the transcript levels of marker genes related to abiotic stress before or after high‐salinity stress (Figure 9). Three kinds of genes have been selected: antiporters (*NHX1* and *SOS1*) or enzyme genes (*P5CS*), negative regulator gene (*SPY‐like*), and DREB transcript factor genes (*DDF1‐like* and *DREB1A‐G*). In transgenic rice plants (Z33 and Z38), almost all selected genes were expressed at higher levels than that in the WT plants under normal condition. After treated with NaCl stress, all of them were expressed highly in both WT and transgenic plants, indicating that these genes are high‐salinity‐responsive. Among them, the expression of *SOS1* gene, which encodes an Na^+^/H^+^ antiporter pumping Na^+^ out of the plant cell from cytosol, was significantly enhanced in NaCl‐treated transgenic lines compared with the WT plants, suggesting that one of functions of PtCYP714A3 is promoting the Na^+^ efflux in transgenic rice, although the exact mechanism that how PtCYP714A3 affects the expression of *SOS1* gene still remains to be clarified. Another intriguing observation is the expression patterns of dehydration‐responsive element‐binding protein 1 (DREB1s). In *Arabidopsis*, DREB1A, DREB1B, DREB1C, DREB2A, and DREB2B proteins are probably the major transcription factors that function in cold‐, high‐salt‐ and drought‐inducible gene expression (Dubouzet *et al*., 2003). In rice, ten putative *DREB1* homologues (Os*DREB1A* to *OsDREB1J*) have been identified and several of them were induced by cold, drought or salinity stress (Dubouzet *et al*., 2003; Mao and Chen, 2012; Wang *et al*., 2008). As *DREB* genes were responsive to drought, we also examined the dehydration tolerance of *PtCYP714A3* transgenic plants (Figure S3b,c). After PEG treatment for 20 days, growth inhibitions were observed in both WT and transgenic rice. Following the recovery without PEG for 2 weeks, transgenic line Z38 grew quite better than did Z33 (Figure S3b). The percentage of biomass with and without PEG treatment (relative biomass) showed that, under osmotic stress condition, the relative biomass ratio in shoots of line Z38 was about 70% of the control, obviously higher than that of the WT plants (49.05%) and transgenic line Z33 (36.88%). These results suggest that although PtCYP714A3 has function on enhancing salt stress tolerance in transgenic rice, it might have no relevant to dehydration resistance of plants.

Semi‐dwarfism has been described as ‘Green Revolution’ morphological phenotype for more resistant to wind and rain damage (Sakamoto, 2006; Sakamoto *et al*., 2003). Previous report has shown that *AtCYP714A2*‐expressing rice produced more yielding tillers and resulted in higher grain productivity than did the wild‐type rice, suggesting a favourable approach for molecular designing of crops with higher grain yield (Zhang *et al*., 2011). To explore the potential of *PtCYP714A3* gene, we compared the yield of wild‐type and transgenic plants grown in the field with or without high‐salinity stress (Table S2). Compared with the wild‐type control, the trait of increased tiller numbers for transgenic plants was not affected by high‐salt stress. In addition, although under both normal and salt stress conditions, the 100‐grain weight and plot yield of the transgenic plants were lower than the wild‐type control, the reductions of 100‐grain weight caused by high‐salinity stress in the transgenic plants were 1.04% (Z33) and 1.92% (Z38), much lower than that of in the wild‐type control (6.23%). All these results suggest that *PtCYP714A3* could be used as an effective gene for engineering transgenic rice with improved salt resistance. The undesirable affects in grain size and plot yield could be due to the unsuitable *OsCYP714D1/Eui* promoter used in this study, as *OsCYP714D1/Eui* was strongly expressed in young panicle and flowering panicle during the heading stage (Zhu *et al*., 2006). Therefore, more suitable promoters such as the *OsGA3ox2* gene promoter could be tried in the future work to avoid the influence of *PtCYP714A3* expression on flower and grain development in transgenic plants (Sakamoto *et al*., 2003; Zhang *et al*., 2011). Taken together, our data suggest that PtCYP714A3 could function in one or several pathway(s) by affecting GA biosynthesis and metabolism. Although the precise mode of the action of PtCYP714A3 in plant growth and response to abiotic stress is still intangible, the results of our study provide direct evidence that altered expression of PtCYP714A3 can significantly modify GA biosynthesis, growth and salt resistance in transgenic plants.

## Experimental procedures

### Plant materials and growth conditions

*Populus trichocarpa* (Torr. & Gray) genotype Nisqually‐1 and a commercial hybrid clone Shanxin yang (*P. davidiana* Dode × *P. bolleana* Lauche) were used in this study. Plants were subcultured on MS medium (Murashige and Skoog, 1962) supplemented with 0.1 mg/L naphthalene acetic acid (NAA). Plants were also grown in the greenhouse under a 12‐h light/12‐h dark photoperiod at 20–25 °C.

Wild‐type rice (*Oryza sativa*) Zhonghua 11 (ZH11) and the *eui* mutant were grown under field conditions or in a greenhouse with a 16‐h/8‐h light and dark photoperiod at 28–30 °C.

### Gene isolation, vector construction and plant transformation

*PtCYP714A3* was cloned into the *Sma* I site in pBlueScript II KS (pKS, Stratagene, La Jolla, CA) for sequence confirmation. For *PtCYP714A3* promoter–GUS construction, the 5'‐flanking DNA (2046 bp) of the *PtCYP714A3* coding region was cloned into the pCAMBIA1300 + pBI101 vector (Liu *et al*., 2003).

For rice transformation, a 2.5‐kb fragment containing the promoter region of *Eui* was cloned into the *EcoR* V site in pKS for sequence confirmation, then digested with *Pst* I and *Bgl* II, and cloned into pCAMBIA1301 to replace the original *CaMV* 35S promoter. To construct the expression vector of *ProEui::PtCYP714A3*, *PtCYP714A3* was placed downstream of the *Eui* promoter in p1301‐ProEui. For the construction of the control expression vector *ProEui::OsCYP714D1, CYP714D1/Eui* cDNA was also cut off with *Bam*H I and *Kpn* I from the vector 35S‐C1301 (Zhu *et al*., 2006) and put downstream of the *Eui* promoter. *ProEui::PtCYP714A3* and *ProEui::OsCYP714D1* were separately transformed into wild‐type ZH11 and *eui* mutant to generate independent transgenic and complementary plants, respectively, as described previously (Hiei *et al*., 1994). T_3_ or T_4_ generations of *PtCYP714A3*‐expressing plants were used for phenotypic analyses. Plant height and other agronomic traits were compared upon maturing with 30 plants for each line.

For poplar transformation, the relative vector was transformed into Shanxin yang as described previously (Wang *et al*., 2011).

### β‐galactosidase (GUS) expression analyses

For histochemical GUS activity assays, the whole plantlet or hand‐cut sections of the stem from three‐week‐old wild‐type and transgenic plants were stained as described previously (Gallagher, 1992).

### Subcellular localization analyses

To determine the subcellular localization of PtCYP714A3‐YFP fusion protein, the encoding region without the stop codon of *PtCYP714A3* was fused in‐frame to the N‐terminal of yellow fluorescent protein (YFP) via the *Xho* I*/Spe* I sites in the pA7‐YFP vector and transfected into the mesophyll protoplasts of Shanxin yang by polyethylene glycol (PEG)‐mediated transfection as described previously (Yoo *et al*., 2007). pA7‐YFP was used as a positive control. For colocalization, the encoding region without the start codon of HDEL, an endoplasmic reticulum (ER)‐localized marker protein (Dong *et al*., 2008), was amplified from *Arabidopsis* cDNA, digested with *Bam*H I/*Spe* I and cloned into the pA7‐CFP vector. After transfected with plasmid DNA, the protoplasts were incubated at 23 °C for 16 h and then examined using a confocal laser scanning microscope (Zeiss LSM 510, Oberkochen, Germany). The excitation wavelengths for YFP and CFP were 514 and 433 nm, respectively.

### PCR and reverse transcriptase (RT)‐PCR analyses

For PCR analyses, genomic DNA was isolated from fresh leaves (about 500 mg for each sample) of WT and transgenic plants cultured in greenhouse as described previously (Kang *et al*., 2010). Gene‐specific primers (Table S1) and GC buffer (TaKaRa, Dalian, China) were used to amplify a 530‐bp PCR product.

For RT‐PCR analyses, total RNA was isolated with the RNAiso Reagent (TaKaRa, Japan) from leaves (for *Populus*) or stems (for rice) of WT and transgenic plants cultured in the greenhouse for 1 month (*Populus*) or 2.5 months (rice). After treated with DNase I (Promega, Madison, USA), 2 μg of total RNA was subjected to reverse transcription reaction using a RevertAid First Strand cDNA Synthesis Kit (Thermo Scientific, Burlington, Canada) at 42 °C for 1 h. GC buffer and gene‐specific primers were the same as used for PCR analysis (30 cycles). The elongation factor gene *PtEF1*β (for *Populus*) and ubiquitin gene *Ubi_1* (for rice) were employed as internal controls with the primers shown in Table S1.

### Quantitative real‐time (qRT)‐PCR

For qRT‐PCR analyses, total RNA was extracted from different organs and tissues of *Populus* or rice as needed, and subjected to reverse transcriptions using a RevertAid First Strand cDNA Synthesis Kit (Thermo Scientific) at 42 °C for 1 h. qRT‐PCR was performed on an AceQ qPCR SYBR Green Master Mix (Vazyme Biotech, Nanjing, China) using a CFX Connect Real‐Time System (Bio‐Rad, Hercules, California) with gene‐specific primers (Table S1). The log_2_ fold change in value was calculated based on 2^−▵Ct^ method. The relative expression of each target gene was normalized using the housekeeping gene *OsUbi‐1*(for rice) or *PtEF1*β (for *Populus*). The error bars were calculated from three biological replicates, and the experiment was repeated at least two times. For expression pattern analysis, the expression value of *PtCYP714A3* in root was set to 1. For the expression level analyses of the selected marker genes, the expression value of wild type was set to 1. For the response analyses of *PtCYP714A3* to high salinity, the expression of the gene under nonsalt stress condition was set to 1.

### GA content determination

Sampled rice stems from 2.5‐month‐old plants (mainly including nodes and internodes) were homogenized in liquid nitrogen using a mortar and pestle, and then lyophilized. An amount of 0.5 g dry weight (DW) of each sample was purified and analysed as described previously (Chen *et al*., 2011).

### Salt stress treatment

To analyse whether the expression of *PtCYP714A3* gene is inducible by salt and other abiotic stresses, leaves of Shanxin yang were cut into pieces (about 1 cm^2^ each piece) and treated with 150 mm NaCl for 0, 3, 6, 12, 18 and 24 h.

For salt tolerance tests, rice seeds were sown in a 96‐well plate (bottom removed). The plate was floated on water and placed to a growth chamber with a 13‐h light (28 °C)/11‐h dark (26 °C) photoperiod. Five days later, the seedlings were cultured with Yoshida's culture solution (Yoshida *et al*., 1976) and the solution was refreshed every 3 days. For salt treatment, three‐week‐old seedlings were transferred to Yoshida's culture solution supplemented with 150 mm NaCl. The solutions were changed every 3 days. The survival rates were counted 10 days after recovering. Plants were also grown in Binhai (Jiangsu Province, China) for field trial in 2013 (transgenic trial permit number: 2013‐T018).

### Na^+^ and K^+^ content assays

After salt treatments, plant materials were harvested and pooled as roots and shoots. The samples were dried for 48 h at 80 °C, milled to fine powder, weighed and digested with concentrated HNO_3_ at 90 °C for 1–2 h. The concentrations of Na^+^ and K^+^ were determined in the digested liquid using an atomic absorption spectrophotometer as described previously (Hitachi Z‐8000, Tokyo, Japan; Wang and Zhao, 1995).

### Statistical analysis

For statistical analyses, the Student's *t*‐test was used to generate every *P* value. The tests were one‐tailed. All data in this work were obtained from at least three independent experiments with three replicates each.

## Supporting information


**Figure S1** Molecular identification and phenotypes of transgenic plants.

**Figure S2** Molecular confirmation and phenotype analyses of transgenic Shanxin yang overexpression of *PtCYP714A3*.

**Figure S3** Expression of *PtCYP714A3* gene in response to PEG treatment and osmotic stress analyses of wild‐type and transgenic plants.

**Table S1** Primers used in this study.
**Table S2** Mean comparisons for tillers and yields of field‐grown wild‐type and PtCYP714A3 transgenic rice plants with or without high‐salinity stress.
