# Supplementary figures and images for: Ectopic expression of a cytochrome P450 monooxygenase gene PtCYP714A3 from Populus trichocarpa reduces shoot growth and improves tolerance to salt stress in transgenic rice
Source: Plant Biotechnol J. 2016 Mar 11;14(9):1838–51. doi: 10.1111/pbi.12544 (PMC5069455; doi:10.1111/pbi.12544)

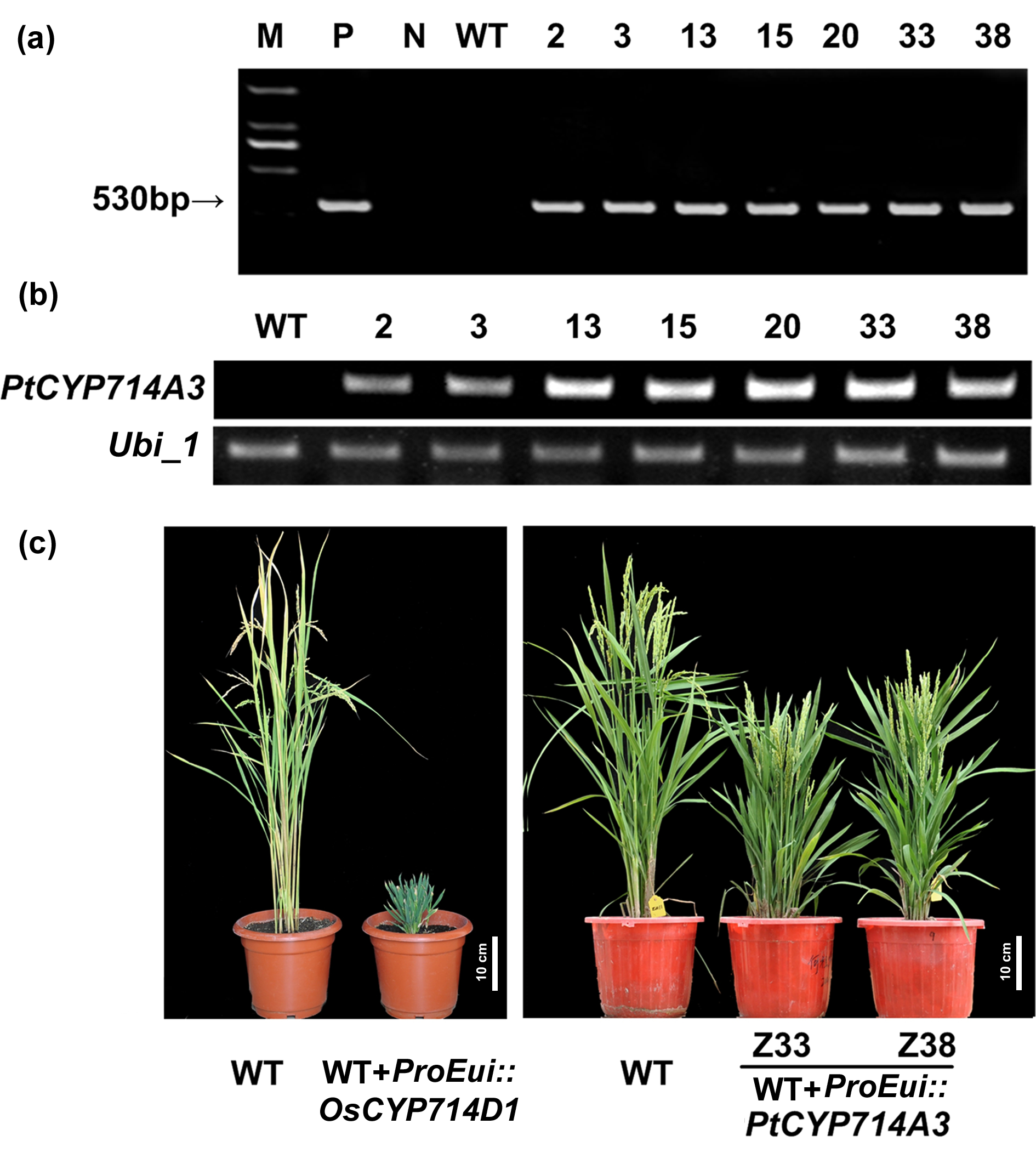

Supplement: Supplementary file 1 — Figure S1 Molecular identification and phenotypes of transgenic plants. [file PBI-14-1838-s003.tiff]

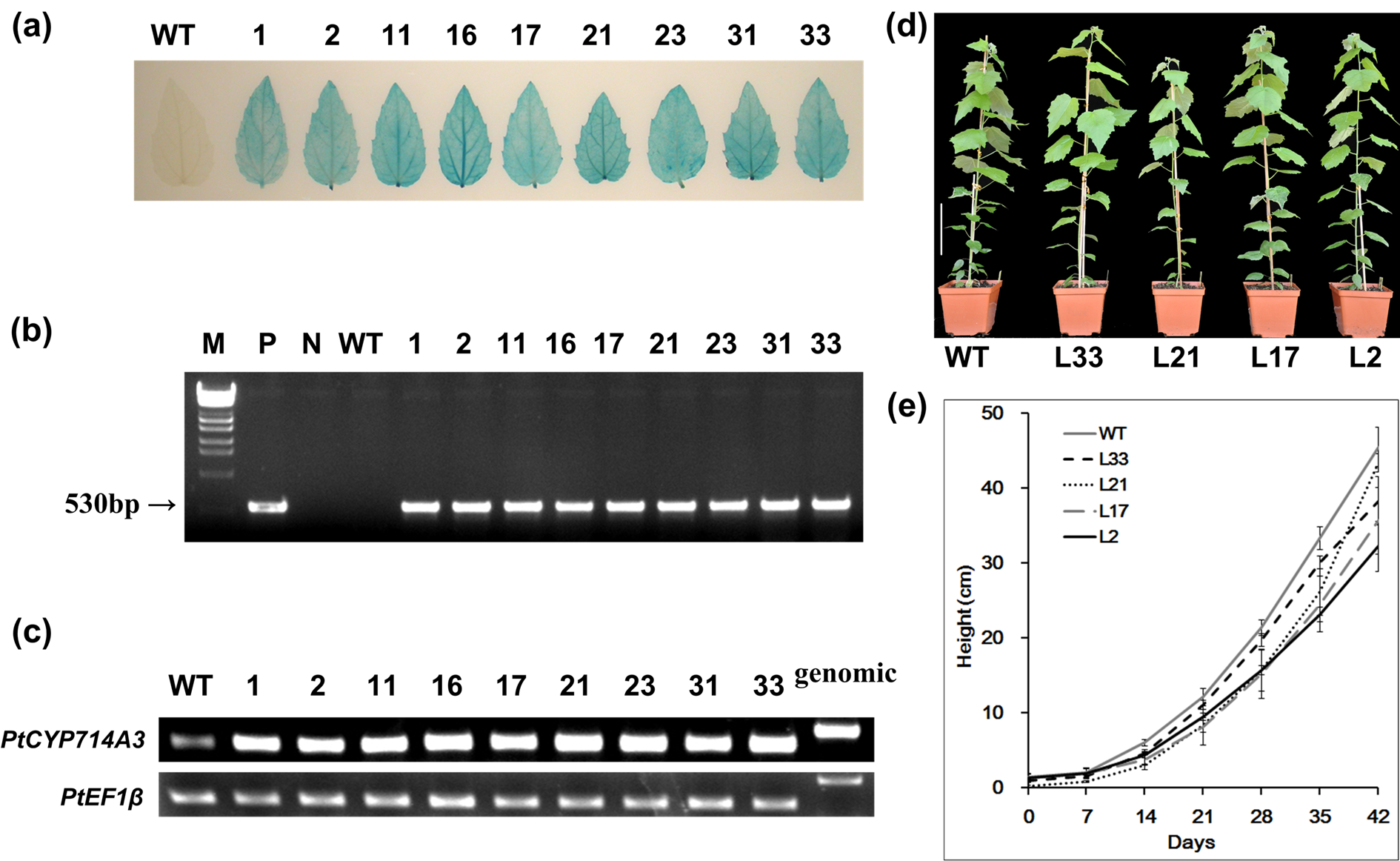

Supplement: Supplementary file 2 — Figure S2 Molecular confirmation and phenotype analyses of transgenic Shanxin yang overexpression of PtCYP714A3. [file PBI-14-1838-s002.tiff]

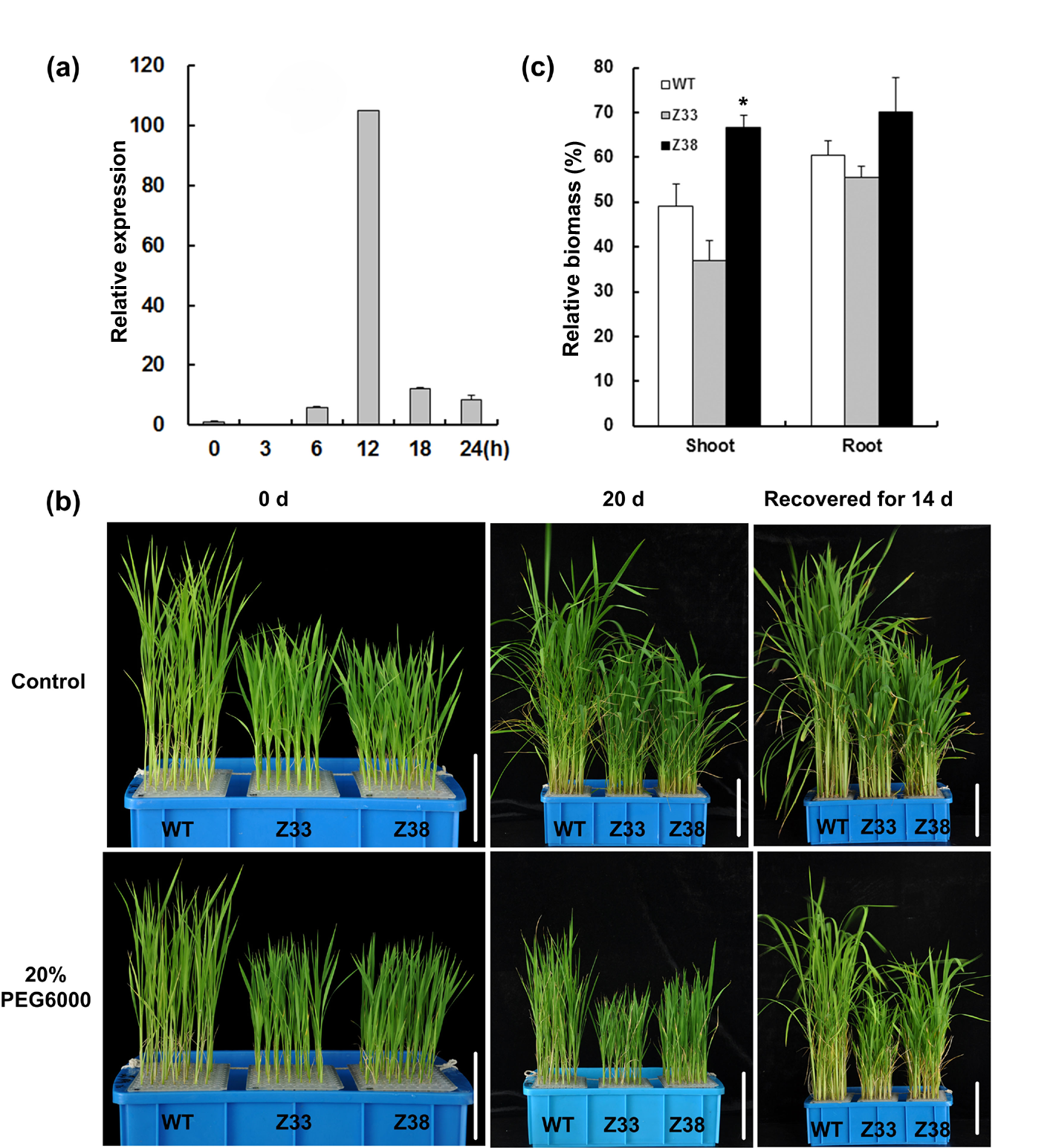

Supplement: Supplementary file 3 — Figure S3 Expression of PtCYP714A3 gene in response to PEG treatment and osmotic stress analyses of wild‐type and transgenic plants. [file PBI-14-1838-s001.tiff]
