# Supplementary material for: Ectopic expression of a cytochrome P450 monooxygenase gene PtCYP714A3 from Populus trichocarpa reduces shoot growth and improves tolerance to salt stress in transgenic rice
Source: Plant Biotechnol J. 2016 Mar 11;14(9):1838–51. doi: 10.1111/pbi.12544 (PMC5069455; doi:10.1111/pbi.12544)
Supplement: Supplementary file 4 — Table S1 Primers used in this study. Table S2 Mean comparisons for tillers and yields of field‐grown wild‐type and PtCYP714A3 transgenic rice plants with or without high‐salinity stress. [file PBI-14-1838-s004.doc]

**Supporting Information**

**Figure S1** Molecular identification and phenotypes of transgenic plants. (a) PCR analyses of *PtCYP714A3* transgenic lines. M, DL2000 DNA marker; P, positive control (plasmid); N, negative control without DNA as template; WT, wild-type; 2, 3, 13, 15, 20, 33 and 38, independently generated transgenic lines. (b) RT-PCR analyses of expression of *PtCYP714A3* gene in transgenic plants. Total RNA was isolated from the stems of WT and transgenic plants cultured in the greenhouse for two and half months. After treated with DNase I (Promega), an amount of 2 μg total RNA was subjected to reverse transcription reaction using a RevertAid First Strand cDNA Synthesis Kit (Thermo Scientific, USA) at 42 °C for 1 h. The resultant cDNA was then used for RT-PCR analyses with GC buffer (TaKaRa, Japan) and gene-specific primers (30 cycles). The ubiquitin gene *Ubi_1* was employed as an internal control (25 cycles). (c-d) Severely dwarfish phenotype of *OsCYP714D1* transgenic (c) and semi-dwarfish phenotype of *PtCYP714A3* transgenic (d) rice plants in comparison with WT.

**Figure S2** Molecular confirmation and phenotype analyses of transgenic Shanxin yang overexpression of *PtCYP714A3*. (a-c) GUS staining, PCR and RT-PCR analyses of transgenic *PtCYP714A3* transgenic *populus*. M, λ-EcoT14 digested DNA markers; P, positive control (plasmid); N, negative control without DNA as template; WT, wild-type; 1-33, independently generated transgenic lines. The elongation factor gene *PtEF1β* was employed as an internal control. (d) Phenotype of WT and transgenic plants cultured in the greenhouse for 42 days. (e) Growth rates of WT and transgenic plants cultivated in the greenhouse. Values are means ±SD from 10 individual plants with three independent biological replicates.

**Figure S3** Expression of *PtCYP714A3* gene in response to PEG treatment and osmotic stress analyses of wild-type and transgenic plants. (a) Expression of *PtCYP714A3* is responsive to PEG stress. Poplar leaves were cut into sections and divided into several groups that were treated separately with 20% (g/v) PEG6000 solution for different time. Samples were collected at 0, 3, 6, 12, 18 and 24 h after the initiation of treatment. Total RNA was extracted and quantitative real-time PCR were performed with *PtCYP714A3* gene-specific primers. The relative expression was normalized by *PtEF1β* and relative to the control value measured at 0 h. Data represent the average of three independent experiments. (b) Phenotype of three-week-old seedlings of the wild-type and *PtCYP714A3* transgenic plants treated with 20% (g/v) PEG6000 solution for 12 d followed by 10 days of recovery culture without PEG stress. WT, wild-type; Z33 and Z38, independent transgenic lines. (c) Relative biomass of transgenic plants compared to the wild-type (WT). After recovery, the PEG-treated plant materials and the control (without PEG treatment) were divided into roots and shoots group, and weighted separately. The FW (fresh weight) ratio of PEG-treated sample to its control was recorded as relative biomass. Values are means ±SD (n=30) from three independent experiments. Asterisks indicate statistically significant difference in comparison to the WT (Student’s *t-test*, *, P<0.05).

**Table S1 Primers used for this article**

| **Primer Name** | | **Sequence** |  |
| --- | --- | --- | --- |
| CYP714A3-F | | 5'ATGGAGGTCTCTCTTCCTTGCC 3' |  |
| CYP714A3-R | | 5'TGGTCAAATCTTTTGAATGAGAATC 3' |  |
| Lox714A3-F | | 5'GCCTCGAGATGGAGGTCTCTCTTCCT 3' |  |
| Lox714A3-R | | 5'GCACTAGTAATCTTTTGAATGAGAATCT 3' |  |
| RT-714A3-F | | 5'TGCCTGCGACTGGTTTCTTCTCT 3' |  |
| RT-714A3-R | | 5'GCTCCGGCTGATTTACATACAAG 3' |  |
| PtEF1β-F | | 5'-GACAAGAAGGCAGCGGAGGAGAG-3' |  |
| PtEF1β-R | | 5'-CAATGAGGGAATCCACTGACACAAG-3' |  |
| RT-Eui-F | | 5'-CGCGGGCTTGCTTTGGGAGTGA-3' |  |
| RT-Eui-R | | 5'-CCGCCGCAGACCTCGAGCACCT-3' |  |
| proEui-F | | 5'-GCCTGCAGTCACGTACACGCATGCCCACCT-3' |  |
| proEui-R | | 5'-GCAGATCTTGGCAGCCTACTCTCTCTTTCC-3' |  |
| pro714A3-F | | 5'GGCTAACCCGTAAAACTTGTGA 3' |  |
| pro714A3-R | | 5'TTTCTTCCACTCTAATGCTGCA 3' |  |
| OsUbi_1-F | | 5'GACGGACGCACCCTGGCTGACTAC 3' |  |
| OsUbi_1-R | | 5'TGCTGCCAATTACCATATACCACGAC 3' |  |
| OsCPS1 -F | | 5'TCGGTTGGAGCGTCTTGGAATA 3' |  |
| OsCPS1 -R | | 5'CGTGGCAAGCTCGCGTACC 3' |  |
| OsKS1-F | | 5'TCCCCACTGAATTGTCCGAAGC 3' |  |
| OsKS1 -R | | 5'TGCCATTCAGCCTCGATCATCAT 3' |  |
| OsKO2 -F | | 5'CGCGCGGAGAGGCAAGGATAT 3' |  |
| OsKO2 -R | | 5'CGCCGCACACCTCTCGAATCTC 3' |  |
| OsKAO -F | | 5'CGGCCCCAAATCCTTCGTCAA 3' |  |
| OsKAO -R | | 5'AGCCCTGAGCGCGCGGTAGTA 3' |  |
| OsGA20ox2-F | | 5'CGCCCACGCCGACCGCTTC 3' |  |
| OsGA20ox2-R | | 5'GCCGCTCCCGCCGCTGGTT 3' |  |
| OsGA3ox2-F | | 5'CGCGCACACGGACTCGGGCTTC 3' |  |
| OsGA3ox2-R | | 5'CGGCCACGTGACAGCGCGGTAG 3' |  |
| OsGA2ox3-F | | 5'CCCGCCCGCCGTCGATCACA 3' |  |
| OsGA2ox3-R | | 5'GCCCCTCCGACATCGCCTCCAT 3' |  |
| OsGID1-F | | 5'-GAGGTCAACCGCAACGAGTGC-3' |  |
| OsGID1-R | | 5'-GCTGCCGCCGTGGAAGAATA-3' |  |
| OsGID2-F | | 5'-CGGGGAGGACCTGGTGTTCG-3' |  |
| OsGID2-R | | 5'-CCCCTCCATTCTTATCACTGTCATTT-3' |  |
| OsNHX1-F | | 5'CGCGGCTGGGGGCTCTGTAC 3' |  |
| OsNHX1-R | | 5'TCCCGACGGCTCCAAATAATGTG 3' |  |
| OsSOS1-F | | 5'TTGGGCGGGACTGCGAGGG 3' |  |
| OsSOS1-R | | 5'GCACAGGCTCATCGTCCAAGTCG 3' |  |
| OsP5CS-F | | 5'TGGGGCGACAGCGACTTAGGTA 3' |  |
| OsP5CS-R | | 5'CAACCCATCCACATCACTGAGCA 3' |  |
| OsSPY-F | | 5'GGGGCGACCGGGGATGGACT 3' |  |
| OsSPY-R | | 5'CATGCATTCCCTGGGTCGATCC 3' |  |
| OsDREB1A-F | | 5'GGCGGAGCGGCAGCACCA 3' |  |
| OsDREB1A-R | | 5'CGGCGAGGGTGCGGTAGGAG 3' |  |
| OsDREB1F_F | | 5'CGCAAGACCGGCCCAAGAGCT 3' |  |
| OsDREB1F_R | | 5'GCGCGTTCCGGCCCTTGAT 3' |  |
| OsDREB2A-F | 5' CGCCAGCCAAGGGGTCCAAGA 3' | | |
| OsDREB2A-R | 5' ATTGCCCTTGCCGCCTCATCGT 3' | | |

**Table S2** Mean comparisons for tillers and yields of field grown wild-type and PtCYP714A3 trangenic rice plants with or without high salinity stress

|  | **Line** | **Tillers per plant** | **Yield per plant (g)** | **100-grain weight**  **(g)** | **Reduction rate of 100-grain weight (%)** | **Yield per plot (g)** | **Yield reduction rate (%)** |
| --- | --- | --- | --- | --- | --- | --- | --- |
| Control | WT | 13.38±1.65 | 17.32±2.52 | 2.46±0.02 |  | 370.31±19.61 |  |
|  | Z33 | 15.13±1.17  * | 9.83±1.76  *** | 2.24±0.03  *** |  | 121.38±4.80  *** |  |
|  | Z38 | 15.50±1.22  * | 10.76±2.99  *** | 2.30±0.01  *** |  | 182.07±14.89  *** |  |
| NaCl | WT | 12.00±2.45 | 10.23±2.66 | 2.31±0.03 | 6.23±1.22 | 243.02±32.23 | 34.37±8.70 |
|  | Z33 | 15.88±2.03  ** | 4.14±3.96  ** | 2.22±0.04  ** | 1.04±1.92  *** | 63.66±3.09  ** | 47.55±2.55 |
|  | Z38 | 17.25±1.71  *** | 5.95±1.74  ** | 2.26±0.04  * | 1.92±1.73  ** | 118.16±20.58  ** | 35.10±11.30 |

Data are from plants in randomized complete block design with three replications grown under natural conditions in Binhai (Jiangsu Province, China) in 2013. Thirty uniformly germinated seeds from wild-type (WT) and homozygous (T_4_ generations) transgenic lines (Z33 and Z38) were sown in a double-row plot, respectively. The plants treated with high salinity were segregated from the control plants with the native rice. High salinity was performed by irrigating one time with the rive water connected to the Yellow Sea (containing about 3.5% salinity) every two normal watering with freshwater. The control plants were irrigated with freshwater at the same time as that of the high salt stress plants. Values are means ±SD from 30 individual plants with three independent biological replicates
